# Frozen and Canned Produce Use and WIC Cash-Value Benefit Redemption in a Tribal Organization

**DOI:** 10.3390/ijerph23060754

**Published:** 2026-06-04

**Authors:** Emily M. Melnick, Francesco Acciai, Nicole Vaudrin O’Reilly, Mindy Jossefides, Punam Ohri-Vachaspati

**Affiliations:** 1 Department of Psychology, University of Denver, Denver, CO 80210, USA; 2 College of Health Solutions, Arizona State University, Phoenix, AZ 85004, USA; facciai@asu.edu (F.A.); pohrivac@asu.edu (P.O.-V.); 3 Inter Tribal Council of Arizona, Inc., Phoenix, AZ 85004, USAmindy.jossefides@itcaonline.com (M.J.)

**Keywords:** WIC, cash-value benefit for fruits and vegetables, American Indian

## Abstract

**Highlights:**

**Public health relevance—How does this work relate to a public health issue?**

- This study addresses the public health issue of maternal and child nutrition among American Indian households.
- Participation in the Special Supplemental Nutrition Program for Women, Infants, and Children (WIC) improves public health nutrition for participating families; however, health benefits depend on redemption of issued food packages.

**Public health significance—Why is this work of significance to public health?**

- Findings show that allowing households to purchase frozen and canned foods using issued WIC cash-value benefits for fruits and vegetables, instead of only fresh, supports benefit redemption among American Indian households in Arizona.

**Public health implications—What are the key implications or messages for practitioners, policy makers and/or researchers in public health?**

- WIC program policies that allow and promote frozen and canned produce options may support benefit use among American Indian families.

**Abstract:**

The Special Supplemental Nutrition Program for Women, Infants, and Children (WIC) provides monthly cash-value benefits (CVBs) for fruits and vegetables. In addition to fresh produce, WIC agencies may allow households to purchase frozen and canned produce using CVBs. The use of these options may support benefit redemption for households who face inequitable barriers to accessing fresh produce, such as households living on tribal lands. This study examined (1) associations between frozen/canned food CVB purchases and overall CVB redemption and (2) predictors of frozen/canned food purchasing within a Tribal Organization using administrative data collected between November 2024 and April 2025 and a participant survey. Administrative data analyses included 4787 Inter Tribal Council of Arizona WIC-participating households; survey analyses included 1165 respondents. Mixed-effects models showed that households purchasing frozen and canned foods using CVBs, instead of only fresh, redeemed more of their CVBs. Further, higher frozen/canned purchasing predicted higher redemption rates. Households with multiple WIC participants were more likely to purchase frozen and canned foods using CVBs than single-participant households. The most commonly reported reason for purchasing frozen/canned foods in surveys was longer shelf life. Findings indicate that allowing and promoting frozen and canned food options may improve CVB utilization for American Indian families.

## 1. Introduction

The Special Supplemental Nutrition Program for Women, Infants, and Children (WIC) offers critical nutrition support to participating pregnant and postpartum mothers, infants, and children under five years of age by providing nutritious tailored food packages to participating households each month. A vital component of the WIC monthly food benefit package for child, pregnant, and postpartum beneficiaries is a cash-value benefit (CVB) for purchasing fruits and vegetables, which currently ranges from $26–52, depending on participant category [1]. Because diets rich in fruits and vegetables promote healthy growth and reduce chronic disease risk [2,3] and because many young children and pregnant and postpartum women do not meet recommendations for fruit and vegetable intake [4,5,6], this benefit has substantial public health relevance. By increasing access to fruits and vegetables, the CVB has the potential to meaningfully improve diet quality among WIC-participating households.

However, the public health benefits of CVBs are contingent upon households redeeming their issued CVBs. Partial or non-redemption of CVBs is relatively common in WIC-participating households and varies across contexts [7,8,9]. Importantly, households living on tribal lands often experience heightened barriers to accessing high-quality, affordable fruits and vegetables due to historical and persistent inequities in food systems [10,11,12]. These barriers are reflected in previously observed lower CVB redemption rates within tribal reservation-based clinics in Washington State (65% at tribal clinics vs. 81% at non-tribal clinics) [13], as well as prior research demonstrating that WIC-participating households on tribal lands report challenges such as limited availability of high-quality fresh produce and needing to travel long distances to redeem their benefits [14]. Thus, identifying predictors of CVB redemption rates is essential for informing policies and interventions to maximize the public health benefit of WIC participation, especially within WIC organizations serving American Indian families living on tribal lands.

One potential predictor of overall CVB redemption that has not been explored in the current literature is household use of frozen and canned fruit and vegetable redemption options. WIC agencies may allow households to purchase frozen and/or canned fruits and vegetables using their CVB in addition to fresh, though policies vary by state [1]. Obtaining frozen or canned foods with longer shelf lives may represent a more feasible option in remote areas, including tribal lands. However, it remains unknown whether purchasing frozen or canned foods using CVBs predicts higher redemption rates. It is also unknown what types of WIC households are more likely to purchase frozen or canned foods and what motivates them to do so.

Therefore, the objective of this study was to assess whether household patterns of purchasing frozen and canned food using CVBs predicted higher CVB redemption rates within a Tribal WIC state agency utilizing administrative data collected over a 6-month period. We also assessed associations between household characteristics and purchasing of canned or frozen foods using CVBs and examined WIC participants’ reasons for purchasing frozen and canned foods with WIC benefits. We hypothesized that greater use of frozen and canned fruit and vegetable redemption options would be associated with higher overall CVB redemption rates in this population of American Indian households. We also hypothesized that households in rural areas, also participating in SNAP, and with multiple WIC participants would be more likely to purchase frozen and canned foods using CVBs.

## 2. Materials and Methods

### 2.1. Study Design

This study was conducted among households participating in the Inter Tribal Council of Arizona (ITCA) WIC program. ITCA is a non-profit member organization representing 21 tribal nations in Arizona and serving as a WIC state agency with a monthly caseload of about 7000 participants across approximately 4000 households. Most ITCA WIC local agencies (12 out of 13) operate clinics and satellite locations on tribal lands.

To test hypotheses about CVB redemption rates and predictors of frozen and canned fruit and vegetable purchasing using CVBs, we used monthly ITCA WIC administrative data for all active ITCA WIC-participating households between November 2024 and April 2025. Models first tested whether combinations of household purchasing patterns (e.g., fresh only vs. fresh and canned) using CVBs predicted CVB redemption rates. Subsequent models then tested whether the proportion of benefits redeemed as frozen or canned predicted CVB redemption rates. Monthly reports included de-identified household identifiers, which were used to link data across reports and to account for repeated observations of households in the analyses. To gather WIC participant perspectives on frozen and canned food purchasing using CVBs, ITCA WIC distributed an online survey via text message to all active households who provided a valid, text-capable phone number. The survey was administered through SurveyMonkey (SurveyMonkey Inc., San Mateo, CA, USA) and was open for completion over a 6-week period between July and August 2025. The research team received the data collected from ITCA WIC in a completely anonymized fashion; thus, the University of Denver institutional review board approved all procedures and determined that full institutional review board review and written informed consent were not required.

### 2.2. Measures

#### 2.2.1. Benefit Redemption

Monthly ITCA WIC administrative reports provided data on CVB amounts issued and redeemed (in dollars) at the household level. CVB redemption rates were operationalized as the ratio of the benefit amount redeemed to the benefit amount issued that month. Reports also provided data on the sub-categories of fruits and vegetables redeemed (i.e., fresh, frozen, canned). Analyses considered household patterns of benefit redemption as both (a) combinations of frozen, canned, and fresh food purchasing using CVBs, and (b) proportion of CVBs redeemed as frozen/canned foods.

To measure household combinations of food purchasing using CVBs, we first created a dichotomous variable representing whether a household purchased only fresh foods using CVBs in a given month or purchased any frozen or canned foods alongside fresh foods using CVBs in a given month (0 = fresh only, 1 = fresh and frozen or canned). A very small portion of the sample (<0.5%) purchased only frozen or canned foods, without any fresh foods, using CVBs in a given month and were not included in the categorical variable. To test whether combinations of canned or frozen food purchasing differentially predicted redemption rates, we also created a 4-category variable reflecting four combinations of food purchasing using CVBs (0 = fresh only, 1 = fresh and frozen only, 2 = fresh and canned only, 3 = fresh and canned and frozen).

To measure the proportion of CVBs redeemed as frozen, we calculated the dollar amount of CVBs redeemed as frozen out of the total dollar amount of CVBs redeemed. Similarly, to measure the proportion of benefits redeemed as canned, we calculated the dollar amount of CVBs redeemed as canned out of the total dollar amount of CVBs redeemed.

#### 2.2.2. Household Characteristics

Monthly ITCA WIC administrative reports provided data on participant and household characteristics. Household-level variables included the race (American Indian vs. not) and ethnicity (Hispanic vs. not) of the most senior WIC-participating member of the household, household SNAP participation (yes vs. no), the number of WIC-participating infants, children, and women in the household, all coded continuously, as well as urbanicity of the local agency attended by the household (urban vs. rural). The urban/rural designation was based on the ITCA WIC program classification, in which local agencies located within large metropolitan areas (e.g., Phoenix) are classified as urban, whereas all other agencies, all located on tribal land, are classified as rural. An additional variable was calculated based on the number of WIC participants in the household for the purposes of analyses assessing predictors of frozen and canned food purchasing using CVBs (single WIC participant in household vs. multiple WIC participants).

#### 2.2.3. Survey Data Variables

To understand frozen and canned fruit and vegetable purchasing using CVBs, ITCA WIC included three survey items on its annual client satisfaction survey. The first question asked households if they were aware that they could purchase frozen and canned fruits and vegetables using their CVBs. A second question asked households if they had ever used their CVBs to purchase frozen and canned fruits and vegetables. If households responded yes, they were asked to select all reasons why they purchase frozen or canned foods from a list (price, easier to prepare, family preferences, hard to find good quality fresh in store, lasts longer) developed based on feedback from ITCA WIC staff and/or provide another reason in an “other” open-end response option. All participant surveys are completely confidential and not linked to respondents’ names. To further protect confidentiality, ITCA WIC staff removed WIC local agency identifiers from the survey data before sharing it with the study team.

### 2.3. Data Analysis

#### 2.3.1. Frozen/Canned Food Purchasing Using CVBs and Redemption

Population-averaged regression models for panel data were used to examine associations between household patterns of purchasing frozen or canned fruits and vegetables using CVBs and overall CVB redemption rates. Because CVB redemption rates are fractional outcomes (i.e., proportions), thus bounded between zero and one, standard linear models, which assume an unbounded continuous outcome, may not provide an accurate representation of the associations of interest. Therefore, we used mixed-effects generalized linear models for proportional data [15]. Specifically, we used the xtgee command in Stata 16 (StataCorp LLC, College Station, TX, USA) to estimate models for panel data with a binomial distribution, probit link function, and robust standard errors. A random effect for household was included to account for potentially repeated observations of the same households over the study period. Analyses were restricted to households that redeemed at least some portion of their issued CVBs during the study period to allow for meaningful comparisons of whether purchasing any frozen or canned foods was associated with higher redemption compared to purchasing only fresh foods. The final analytic sample included 21,263 observations of 4787 unique households.

The first modeling step tested whether different combinations of household food purchasing using CVBs were associated with overall CVB redemption rates. An initial model tested whether redeeming any frozen or canned foods (vs. only redeeming fresh foods) using CVBs predicted higher overall CVB redemption. This model included a dichotomous food purchasing variable (fresh only, for households that exclusively redeemed fresh foods vs. some frozen and/or canned, for households that redeemed at least some frozen or some canned foods) as the main predictor and overall CVB redemption rate as the outcome. A second model included a 4-category food purchasing variable (fresh only; fresh and frozen only; fresh and canned only; fresh and frozen and canned) as the main predictor and overall CVB redemption rate as the outcome to assess whether specific combinations of frozen and canned food purchasing were differentially associated with CVB redemption rates. A fifth category (frozen and canned only) was considered but dropped from analyses, as it represented less than 0.5% of the sample. Postestimation commands (i.e., margins and lincom) were used to assess differences in predicted CVB redemption rates across household food purchasing combinations.

The second modeling step tested whether the proportion of CVBs redeemed as frozen or canned fruits and vegetables was associated with overall CVB redemption rates. This model included the proportion of CVBs redeemed as frozen foods and the proportion of CVBs redeemed as canned as the two main predictors and overall CVB redemption rate as the outcome. An interaction term between proportion of CVBs redeemed as frozen and the proportion of CVBs redeemed as canned was included to assess whether concurrent use of both options (frozen and canned) was associated with differential redemption rates. We then used postestimation commands (i.e., margins) (1) to determine predicted overall CVB redemption rates at specific levels of frozen and canned food redemption, and (2) to construct the figure presented. Both modeling steps included race and ethnicity of the most senior WIC participant in the household, household composition, and urbanicity of the attended WIC agency as covariates.

#### 2.3.2. Household Characteristics and Frozen/Canned Food Purchasing Using CVBs

To examine associations between household characteristics (SNAP participation, urbanicity, single vs. multiple WIC-participating members, race, and ethnicity of the most senior WIC-participating household member) and use of CVBs for frozen or canned foods purchases, we applied a two-step mixed-effects modeling approach. All models included a random effect for household to account for repeated observations over the study period. In the initial modeling step, we estimated separate bivariate mixed-effects logistic regression models, specifically, one for each association. In the second modeling step, we estimated a multivariable mixed-effects logistic regression model that included all assessed characteristics in the same model. Statistical comparisons for all regression models were considered significant at *p* < 0.05.

Frequencies were calculated for survey items about frozen and canned food purchasing to contextualize and provide additional insights into the model results. For the purposes of survey analyses, the survey sample was restricted to households who had at least one woman or child participant in the household, as these are the participant categories issued CVBs by default as a part of their monthly food benefit package. Survey findings are described in text only in the results section and are not presented in a table or figure.

## 3. Results

### 3.1. Sample Description

Key characteristics of households included in the sample for analyses using administrative data from a snapshot month of November 2024 (*n* = 3899), the first month in the study period, are shown in Table 1. About half of the households reported also participating in SNAP (50.2%), 63.7% were attending a rural WIC local agency, and 39.2% had more than one WIC participant in the household. A total of 1608 respondents completed the household survey, representing about 40% of active ITCA WIC households during the months when the survey was distributed. The analytic sub-sample, including only households that had at least one woman or child in the household, comprised 1194 respondents. Slightly more than half (54%) of respondents in the analytical sub-sample came from households attending rural ITCA WIC local agencies.

### 3.2. Frozen/Canned Food Purchasing Using CVBs and Redemption

Estimates from model 1, using a binary food redemption variable, indicate that households redeeming CVBs for any frozen or canned foods (in combination with fresh foods) redeemed a greater proportion of their issued CVBs compared to households redeeming exclusively fresh foods (B = 0.73, *p* < 0.001, 95% CI: 0.67, 0.78; see Table 2). Model-predicted CVB redemption rates were 78.8% for households redeeming only fresh foods and 88.5% for households redeeming any frozen and/or canned foods in addition to fresh foods.

**Figure 1:**
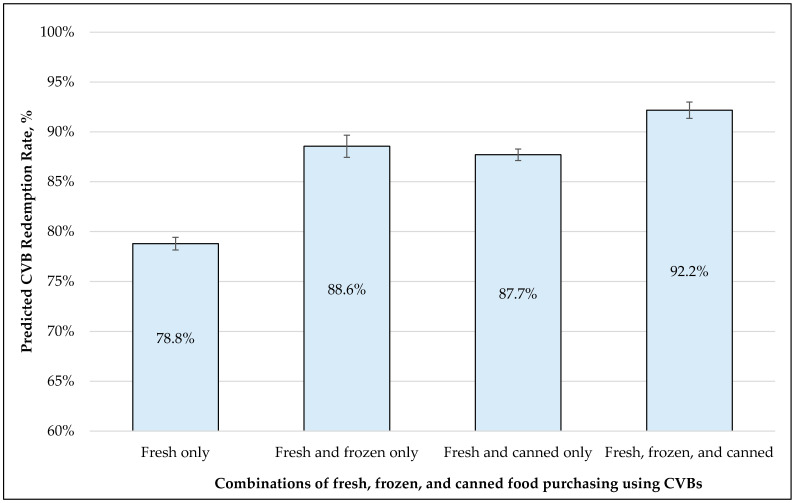
Predicted CVB redemption rate by combinations of fresh, frozen, and canned food purchasing using CVBs among Inter Tribal Council of Arizona WIC-participating households (*n* = 21,263 observations of 4787 unique households over a 6-month period). The model includes the following controls: household SNAP participation (yes vs. no), race (American Indian vs. not) and ethnicity (Hispanic vs. not) of the most senior WIC-participating household member, the number of WIC-participating infants, children, and women in the household, all coded continuously, and urbanicity of the WIC local agency attended by the household (urban vs. rural).

Results from model 2, using a four-category food purchasing variable, showed that households purchasing (a) fresh and frozen, (b) fresh and canned, and (c) fresh, frozen, and canned foods redeemed a significantly greater proportion of their issued CVBs compared to households that purchased fresh foods only (*p* < 0.001 for all comparisons; see Table 2). As shown in Figure 1, model-predicted redemption rates were highest among households that purchased fresh, frozen, and canned foods (92.2%), compared to households that purchased fresh and frozen only (88.6%) and fresh and canned only (87.7%; *p* < 0.001 for both comparisons).

**Figure 2:**
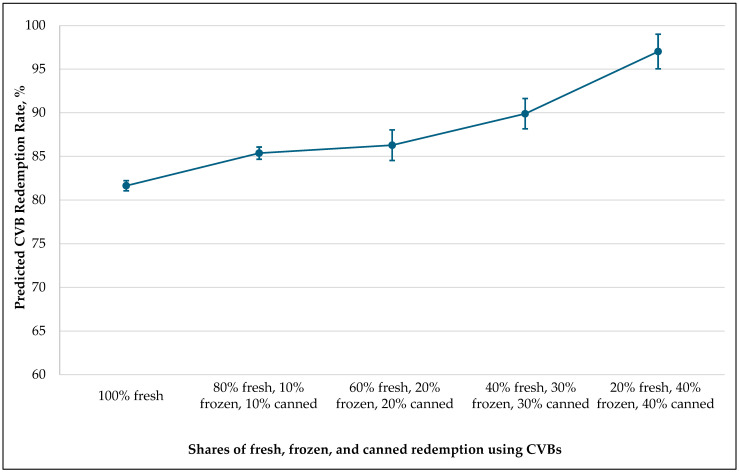
Predicted CVB redemption rate by shares of fresh, frozen, and canned redemption using CVBs among Inter Tribal Council of Arizona WIC-participating households (*n* = 21,263 observations of 4787 unique households over a 6-month period). The model includes the following controls: household SNAP participation (yes vs. no), race (American Indian vs. not) and ethnicity (Hispanic vs. not) of the most senior WIC-participating household member, the number of WIC-participating infants, children, and women in the household, all coded continuously, and urbanicity of the WIC local agency attended by the household (urban vs. rural).

Model estimates from the second modeling step extend the findings that redeeming any frozen/canned foods is associated with higher overall CVB redemption and show a positive association pattern between the proportion of benefits redeemed as frozen/canned and overall CVB redemption (see Table S1 for model coefficients for all predictor variables). As the proportion of benefits redeemed as frozen foods increases, the overall CVB rate tends to increase (B = 1.18, *p* < 0.001, 95% CI: 0.68, 1.68). A similar positive association was observed for the proportion of benefits redeemed as canned foods and overall CVB redemption rate (B = 0.80, *p* < 0.001, 95% CI: 0.52, 1.07). Estimates also reveal that when redemption of frozen and canned foods occurs simultaneously, we observe an additional increase in the overall redemption rate, as indicated by the positive interaction term (B = 7.53, *p* = 0.003, 95% CI: 2.64, 12.41). To facilitate the interpretation of these estimates, Figure 2 shows model-predicted CVB redemption rates for the average household, at different proportions of benefits redeemed as fresh, frozen, and canned.

### 3.3. Household Characteristics and Frozen/Canned Food Purchasing Using CVBs

Table 3 presents results from bivariate and multivariate analyses using administrative data to assess predictors of household purchasing of frozen and canned foods using CVBs. Bivariate model results with frozen food purchasing as the outcome showed that households with multiple WIC participants were more likely to purchase any frozen foods (15.1% vs. 8.6%, *p* < 0.001) using CVBs compared to households with only one WIC participant. Households in urban areas were more likely to purchase any frozen foods using CVBs (13.5% vs. 9.9%, *p* < 0.001) and households participating in SNAP were less likely to purchase any frozen foods using CVBs (12.3% vs. 10.0%, *p* < 0.0001). Race and ethnicity of the most senior WIC-participating household member did not predict differences in household likelihood to purchase frozen foods using CVBs. All patterns of results for differences between groups remained the same in a multivariate model that included all predictors.

Bivariate model results with canned food purchasing as the outcome showed that households with multiple WIC participants were more likely to purchase any canned foods (44.1% vs. 29.2%, *p* < 0.001) using CVBs compared to households with only one WIC participant. In addition, households in which the most senior WIC-participating household member identified as American Indian were less likely to purchase canned foods compared to non-American Indian households (32.0% vs. 35.5%, *p* = 0.007). Both group differences by WIC-participating household composition and race remained in the multivariate model. In addition, in the multivariate model, households participating in SNAP were less likely to purchase canned foods using CVBs compared to non-participating households (34.4% vs. 36.1%, *p* = 0.033).

Within the survey sample, 73% of respondents (*n* = 872 out of 1194) reported being aware that CVBs could be used to purchase frozen or canned fruits and vegetables. Among those aware of this option, 75% (*n* = 652 out of 872) reported having purchased frozen or canned fruits and vegetables using their CVBs at least once. Among respondents who reported purchasing frozen or canned fruits and vegetables using their CVBs, the most commonly cited reason was longer shelf life (84%). Other reasons included difficulty finding good quality fresh in store (23%), ease of preparation (22%), family preference (22%), and price (22%). The most common written-in response (entered as open-ended text by respondents who selected ‘Other’) was that they prefer to purchase frozen fruits and vegetables to make smoothies (written in by 15 respondents, 2.3% of total responses).

## 4. Discussion

This study provides new evidence to inform WIC policies and programs by examining how purchasing of frozen and canned foods using CVBs is associated with overall fruit and vegetable benefit redemption within a Tribal WIC state agency. The study results indicate that purchasing frozen and canned fruits and vegetables using CVBs is associated with higher overall CVB redemption, consistent with our first hypothesis. Specifically, households that redeem a share of their CVBs as frozen or canned food tend to redeem a larger proportion of their monthly CVBs compared to households that redeem CVBs exclusively for fresh fruits and vegetables. Moreover, the concurrent use of both frozen and canned options was associated with an additional increase in overall redemption, suggesting that combining multiple non-fresh redemption options may further support benefit utilization. We also observed a positive association pattern between frozen and canned food purchases using CVBs and overall CVB redemption, whereby greater use of frozen and/or canned food redemption options was associated with more complete benefit utilization.

Consistent with our second hypothesis, households with multiple WIC participants were more likely to purchase frozen as well as canned foods using their CVBs. Larger households receive higher total CVB amounts, and purchasing foods with longer shelf lives may help households manage benefits more effectively across the month. We also observed that households from urban areas and those not participating in SNAP were more likely to purchase frozen fruits and vegetables. The higher use of frozen food purchasing among urban households may reflect differences in retail infrastructure, including freezer availability, as reported by ITCA WIC staff during a local agency managers meeting in which these findings were presented. These findings highlight the importance of food store capacity—particularly in rural and tribal communities—and suggest that investments in retail infrastructure on tribal lands may improve access to frozen fruits and vegetables for WIC-participating households [13]. Limited freezer availability in some stores in which ITCA WIC households shop may also partly explain why purchasing frozen foods using CVBs was less common than purchasing canned foods using CVBs in this sample. The finding that SNAP participation predicted lower likelihood of purchasing frozen foods and of predicted canned foods in the multivariate model was not in line with study hypotheses. We had hypothesized that SNAP-participating households would be more likely to purchase frozen and canned foods, as households receiving additional food benefits may prefer items with longer shelf lives. Future research is needed to confirm and further explore these findings.

Survey findings further contextualize administrative results by providing insight into why households choose frozen and canned options. Longer shelf life emerged as the primary factor followed by difficulty finding fresh foods in store, ease of preparation, and family preference. These findings align with known access barriers faced by households living in remote areas, including findings showing significant disparities in CVB redemption for WIC clinics based on tribal lands [13,16]. Together, these results reinforce the value of flexible CVB redemption options for supporting benefit utilization in settings where access to fresh produce may be limited. These findings also align with prior qualitative work conducted within a Tribal WIC agency, in which staff reported that households living on tribal lands found frozen and canned produce easier to purchase using CVBs than fresh produce and valued having access to multiple non-fresh options [14].

To fully realize the benefits of these flexibilities, it is essential that families are aware of their ability to purchase frozen and canned foods using CVBs. In our sample, 27% of survey respondents were not aware that CVBs could be used to purchase frozen or canned fruits and vegetables, suggesting opportunities to increase benefit utilization, which may be mirrored in other WIC state and local agencies. Nutrition education and participant communications could more explicitly highlight frozen and canned options and provide practical guidance on their use. For example, several survey respondents reported enjoying frozen options to make smoothies and may appreciate recipes for making healthy smoothies. Prior studies suggest that recipe-based nutrition education featuring WIC-approved foods is well received by participating households [17,18,19].

Notably, policies that allow frozen and canned food redemption vary across WIC state agencies. Current federal guidance requires WIC agencies to authorize at least one non-fresh form of fruits and vegetables (frozen, canned, or dried) in addition to fresh produce [1]. Across both models examining household redemption patterns of CVB utilization, findings showed households that purchased fresh, frozen, and canned foods within a given month were predicted to redeem the highest share of issued CVBs compared with other purchasing combinations. These findings provide supportive evidence for WIC program policies that authorize multiple non-fresh options, rather than a single non-fresh option, to better support household needs and maximize CVB redemption. The findings also suggest that WIC staff guidance encouraging participants to purchase a combination of fresh, frozen, and canned produce options may help households optimize benefit use.

These findings should be considered in the light of several strengths and limitations. Notable strengths include the use of administrative data over an extended period of time for a WIC state agency and the integration of survey data to contextualize household purchasing behaviors. However, representativeness of the household survey sample may be a limitation, as respondents, representing 40% of the active households, may represent those more engaged in the WIC program. In addition, because the survey was distributed via text message, respondents living on tribal lands with limited broadband reception [20] may have been less likely to receive the survey link to participate. Nonetheless, households attending rural ITCA WIC local agencies were only slightly underrepresented in the survey sample (54%), compared to the ITCA WIC caseload for rural agencies for the same period (63%). Methodological limitations should also be considered. Although models accounted for repeated household observations and key covariates, unmeasured factors (e.g., store-level availability, household transportation access, freezer capacity, or preferences for specific food forms) may have influenced purchasing behavior and redemption rates. Additional sources of unmeasured confounding may exist. For example, households that are more familiar with WIC or more motivated to fully utilize benefits may also be more likely to purchase frozen and canned foods. These limitations, along with the observational nature of the study, limit causal interpretation of the findings. The available data aligned with the scope of the study also precluded examination of whether policies permitting multiple types of non-fresh produce facilitate transitions from none to any CVB redemption. Future studies utilizing pre- and post-policy implementation data are warranted to investigate this question. Finally, this study focused on a single Tribal WIC state agency serving primarily American Indian families in Arizona. Thus, the findings reflect unique contextual factors and may not be generalizable to other WIC agencies. Future studies in other WIC agencies are needed to assess broader applicability of these findings.

## 5. Conclusions

In summary, purchasing frozen and canned fruits and vegetables using CVBs, rather than only fresh produce, was associated with higher CVB redemption within a Tribal WIC agency. Additionally, purchasing a combination of fresh, frozen, and canned produce may help households maximize redemption. These findings support WIC policies permitting both frozen and canned produce purchases using CVBs. Findings also suggest that program efforts to improve awareness and promote use of frozen and canned fruits and vegetables are warranted to improve benefit utilization to fully realize the health benefits of WIC participation for American Indian households.

## Figures and Tables

**Table 1:** Characteristics of Inter Tribal Council of Arizona WIC households during November 2024, the first evaluated month of the study period (*n* = 3899 households).

Sample Characteristics	Mean (SD) or % (*n*)
Participating in SNAP, % yes (*n*)	50.2 (1956)
Hispanic, % yes (*n*)	31.2 (1217)
WIC participant composition in household	
Number of WIC-participating children in household, m (SD)	1.00 (0.67)
Number of WIC-participating women in household, m (SD)	0.28 (0.45)
Number of WIC-participating infants in household, m (SD)	0.25 (0.44)
Total number of WIC participants in household, m (SD)	1.53 (0.75)
More than one WIC participant in household, % (*n*)	39.9 (1554)
Attending WIC local agency in rural area, % (*n*)	63.7 (2483)
CVB redemption rate, mean % (SD)	72.2 (35.37)

**Table 2:** Results from mixed-effects generalized linear models testing associations between household combinations of purchasing fresh, frozen, and canned foods and CVB redemption rates among Inter Tribal Council of Arizona WIC participating households (*n* = 21,263 observations of 4787 unique households over a 6-month period) ^1^.

	Model 1				Model 2			
Predictor Variable	Coef.	*p*-Value	95% CI		Coef.	*p*-Value	95% CI	
			Lower Limit	Upper Limit			Lower Limit	Upper Limit
Two-category combination variable (ref: fresh only)								
Fresh and frozen or canned	0.73	<0.001	0.67	0.78				
Four-category combination variable (ref: fresh only)								
Fresh and frozen only					0.74	<0.001	0.63	0.85
Fresh and canned only					0.66	<0.001	0.60	0.71
Fresh, frozen, and canned					1.16	<0.001	1.04	1.28

^1^ Models include the following controls: Household SNAP participation (yes vs. no), race (American Indian vs. not) and ethnicity (Hispanic vs. not) of the most senior WIC-participating household member, the number of WIC-participating infants, children, and women in the household, all coded continuously, and urbanicity of the WIC local agency attended by the household (urban vs. rural).

**Table 3:** Results from mixed-effects logistic regression models testing associations between Inter Tribal Council of Arizona WIC-participating household characteristics and purchase of any frozen or canned foods with their cash-value benefit for fruits and vegetables (CVB) among households who redeemed any monthly benefits (*n* = 21,263 observations of 4787 unique households).

	Separate Bivariate Models		Multivariate Models	
Predictor Variable	Model Predicted Mean Likelihood of Purchasing	*p*-Value for Diff. Between Groups	Model Predicted Mean Likelihood of Purchasing	*p*-Value for Diff. Between Groups
Frozen Food Purchasing Using CVBs				
WIC clinic urbanicity				
Urban	13.47		13.34	
Rural	9.88	<0.001	9.94	<0.001
SNAP participation				
Participating	10.02		10.09	
Not participating	12.34	<0.001	12.30	<0.001
WIC Participant composition				
Single participant	8.59		8.53	
Multiple participants	15.14	<0.001	15.19	<0.001
Race				
American Indian	11.29		11.41	
Not American Indian	10.82	0.560	10.16	0.134
Ethnicity				
Hispanic	11.21		10.79	
Not Hispanic	11.25	0.957	11.44	0.308
Canned Food Purchasing Using CVBs				
WIC clinic urbanicity				
Urban	34.89		35.15	
Rural	35.10	0.835	35.33	0.858
SNAP participation				
Not participating	35.45		36.14	
Participating	34.57	0.296	34.36	0.033
WIC Participant composition				
Single participant	29.23		29.19	
Multiple participants	44.10	<0.001	44.17	<0.001
Race				
American Indian	35.51		35.75	
Not American Indian	32.01	0.007	32.34	0.012
Ethnicity				
Hispanic	34.11		34.83	
Not Hispanic	35.45	0.170	35.47	0.544

## Data Availability

The data cannot be shared due to tribal data sovereignty.

## Supplementary Materials

The following supporting information can be downloaded at: https://www.mdpi.com/article/10.3390/ijerph23060754/s1, Table S1: Results from a mixed-effects generalized linear model testing associations between the proportion of CVBs redeemed as frozen or canned foods and overall CVB redemption rates among Inter Tribal Council of Arizona WIC participating households (*n* = 21,263 observations of 4787 unique households over a 6-month period).

## Abbreviations

The following abbreviations are used in this manuscript:

WIC	Special Supplemental Nutrition Program for Women, Infants, and Children
CVB	Cash-Value Benefit for Fruits and Vegetables
