# Supplementary material for: Frozen and Canned Produce Use and WIC Cash-Value Benefit Redemption in a Tribal Organization
Source: Int J Environ Res Public Health. 2026 Jun 4;23(6):754. doi: 10.3390/ijerph23060754 (PMC13299148; doi:10.3390/ijerph23060754)
Supplement: Supplementary file 1 [file ijerph-23-00754-s001.zip › ijerph-4275978-supplementary.pdf]

Supplementary Files

Supplementary Table S1. Results from a mixed-effects generalized linear model testing associations between the proportion of CVBs redeemed as frozen or canned foods and overall CVB redemption rates among Inter Tribal Council of Arizona WIC participating households (n = 21,263 observations of 4,787 unique households over a 6-month period).[1]

**Predictor Variable**	**Coef.**	***P*-value**	**95% CI**	
			Lower limit	Upper limit
% of CVBs redeemed as frozen foods (a)	1.18	<0.001	0.68	1.68
% of CVBs redeemed as canned foods (b)	0.80	<0.001	0.52	1.07
Interaction (a) * (b)	7.53	0.003	2.64	12.41

[1] Model included the following controls: Household SNAP participation (yes vs. no), race (American Indian vs. not) and ethnicity (Hispanic vs. not) of the most senior WIC-participating household member, the number of WIC-participating infants, children, and women in the household, all coded continuously, and urbanicity of the WIC local agency attended by the household (urban vs. rural).
